# Automatisierte, randomisierte Online-Programmierprüfung mit geringem, kurzzeitigem Datenverkehr

**DOI:** 10.1007/s00287-023-01534-y

**Published:** 2023-04-12

**Authors:** Nils Beckmann

**Affiliations:** Fachbereich Elektrotechnik und Technische Informatik, Technische Hochschule Ostwestfalen-Lippe, Campusallee 12, 32657 Lemgo, Deutschland

## Abstract

Die Möglichkeit zur Teilnahme an Online-Prüfungen ist generell bedingt durch die Stabilität und Geschwindigkeit der Internetverbindung der Prüfungsteilnehmer sowie die Zuverlässigkeit der Computer und Server, über welche die Prüfung bereitgestellt wird. Insbesondere bei großen Teilnehmerzahlen können hohe Serverlasten zu Beginn und Ende der Prüfung auftreten, wenn viele Teilnehmer zur selben Zeit größere Dateien herunter- oder hochladen. Im Folgenden wird eine Prüfungsmethode vorgestellt, die diese beiden Hürden für eine Prüfungsteilnahme reduziert. Hierbei sind die zu übertragenden Datenmengen gering, betragen insgesamt weniger als ein halbes Megabyte je Teilnehmer und müssen nur zu Beginn und Ende der Prüfung einmalig übermittelt werden. Die Prüfungsmethode eignet sich insbesondere für Programmierprüfungen, ist aber auch für andere Fachgebiete anwendbar. Es stehen verschiedene Fragentypen zur Auswahl. Die Aufgaben werden zufällig auf die Teilnehmer verteilt, können lokal bearbeitet sowie anschließend größtenteils automatisiert korrigiert und ausgewertet werden.

## Einleitung

Über informationstechnische (IT) Grundfähigkeiten hinaus, die heute in vielen Ausbildungs- und Studiengängen von Relevanz sind [1], gehört auch die Programmierung in einer Programmiersprache insbesondere in den technischen Disziplinen oftmals zum Grundlagenkanon an Wissen und Fähigkeiten [2]. Diese so genannten „Future Skills“ [1] werden bereits an vielen Hochschulen in Deutschland im Rahmen von IT-Studiengängen, wie beispielsweise Informatik, Elektrotechnik, Technische Informatik und so weiter, gelehrt [3]. Zur Überprüfung dieser Fach- und Methodenkompetenzen der Studierenden [4] gibt es an Hochschulen seit Jahrhunderten das Konzept der Prüfung in verschiedenen Formaten (u. a. Klausur, mündliche Prüfung, Präsentation, Kolloquium, schriftliche Ausarbeitung). Durch die Corona-Pandemie (COVID-19) wurde die Notwendigkeit zur Nutzung von IT-Kenntnissen und -Methoden verstärkt [5], nicht nur für Studierende, sondern auch für Lehrende im Rahmen von Lehrveranstaltungen sowie Prüfungen [6]. Eine zentrale Fragestellung ist, ob und inwiefern bisherige Präsenzprüfungsformate in digitale Formate überführt oder neugestaltet werden können. Wichtig ist hierzu, dass technische Hilfsmittel gut vorbereitet und die Studierenden frühzeitig und umfassend in den neuen Ablauf eingeführt werden [7], damit Zugangshürden abgebaut werden und vergleichbare Prüfungsresultate zwischen präsenz‑/papierbasierten Prüfungs- und Klausurformaten sowie digitalen Prüfungsformaten erzielt werden können [8].

Zu diesem Thema werden in diesem Beitrag eine Methode und ihre Anwendung vorgestellt, mit der fragebogenbasierte Klausurprüfungen in Lehrmodulen, insbesondere (aber nicht ausschließlich) mit programmiertechnischem Bezug, digital automatisiert durchgeführt als auch bewertet werden können. Die hier vorgestellte Methode kommt mit niedrigem und nur kurzzeitigem Datenverkehr aus. Damit werden 2 typische Hürden/Probleme von üblichen, digitalen Prüfungsformaten vermieden, bei denen die Teilnehmer permanent über Internetzugriff verfügen müssen und/oder durch den Upload/Download großer Datenmengen erhebliche Serverlasten entstehen (beispielsweise beim Hochladen von Fotos/Scans der schriftlich erstellten Prüfungsantworten; [6]). Hierdurch können auch Studierende mit unzuverlässigen Internetverbindungen an Online-Prüfungen teilnehmen. Darüber hinaus sind durch die hier vorgestellte Methode aufgrund der Automatisierung erhebliche Zeiteinsparungen bei der Bewertung der Prüfungsantworten der Teilnehmer erreichbar. Beispiele für solche Lernplattformen, über die (teil-/automatisiert) Prüfungen abgewickelt werden können, sind die freien Softwares „ILIAS“ [9] oder „Moodle“ [10].

### Rahmenbedingungen

Konkret wurde die Methode erstmals im Wintersemester 2020/2021 an der Technischen Hochschule Ostwestfalen-Lippe (TH OWL) im Fachbereich „Elektrotechnik und Technische Informatik“ im Modul „Programmiersprachen 1“ (1. Fachsemester) mit etwa 200 Studierenden angewandt. Im Kurs sollen die Studierenden (innerhalb von vorgesehenen 150 h Arbeitszeit) die Grundlagen der prozeduralen Programmierung anhand der Programmiersprache C erlernen [11, 12]. Dazu gehören beispielsweise Syntax, Semantik, Schlüsselwörter, Anweisungen, Kontrollstrukturen, Standardbibliotheken und so weiter. Durch je 2 Semesterwochenstunden (SWS) Theorie (Vorlesung) und Praxis (Praktikum) verbessern die Studierenden ihre Fach- und Methodenkompetenz [4, 13]. Die zu erreichenden Lernzielniveaustufen sind das Erinnern, Verstehen und Anwenden [14]. Überprüft wird das Wissen üblicherweise mittels einer Klausurleistung, die innerhalb von 90 durchgängigen Minuten auf Papier schriftlich erbracht werden muss. Aufgabentypen sind vielfältig und umfassen das Schreiben von Quelltexten gemäß Anforderungen, Fehlerfinden und Quelltextkorrektur, Wissens- und Verständnisfragen, Vorhersage von Variableninhalten und Programmausgaben und so weiter. Das vollständige Lernsetting des Moduls ist an anderer Stelle publiziert [15]. Im Zuge der Corona-Pandemie waren Prüfungen in Präsenz (Studierende sind währenddessen vor Ort und werden durch Aufsichtspersonal beaufsichtigt) vielerorts in Deutschland aufgrund politischer Bestimmungen und aus Gründen der Gesundheitsprävention nicht mehr möglich. In den Hochschulen wurde entsprechend reagiert und nicht nur digitale Lehrformate, sondern auch digitale Prüfungsformate wurden zur Regel, so auch an der TH OWL. In diesem Kontext entstand die im Folgenden vorgestellte Methode, die eine Alternative zu anderen bereits vorhandenen Online-Prüfungsformaten darstellt.

### Methode und Prüfungsablauf

Der Rahmen des technischen Ablaufs wird über die o. g. Lernplattform ILIAS mit dem Modul „EAssessment“ vermittelt [9]. Studierende melden sich frühzeitig (optimal mehrere Wochen vorher) in einem extra für die Prüfung erstellten Kurs mit ihren digitalen Hochschulprofilen an und werden, sofern diese regulär über das Prüfungsamt für die Prüfung angemeldet sind, dafür manuell freigeschaltet. Zur Startzeit der Prüfung erhalten die Teilnehmer Zugriff auf die 2 Prüfungsdateien (Prüfungsskript sowie Fragenkatalog) durch Herunterladen (eine Zusammenstellung über alle für die Prüfung notwendigen Dateien vermitteln Tab. 2 und Tab. 3 ganz unten). Das Prüfungsskript ist ein zu kompilierender Quelltext in der Programmiersprache C, durch welchen das Prüfungsprogramm generiert wird. Die Bearbeitungszeit einer Prüfung beträgt üblicherweise 90 min zuzüglich einer digitalen Pufferzeit (üblich sind 30 min im Fachbereich, was für die vorgestellte Prüfungsform recht großzügig ist) für Herunterladen und Hochladen. Vor Ablauf dieser Zeiten müssen die Teilnehmer ihre 2 Abgabedateien (Antwortdatei und Klartextdatei) hochgeladen haben, ansonsten gilt die Prüfung als „nicht bestanden“ bzw. „nicht erschienen“. Der Dateiname der Letzteren beiden besteht aus der jeweiligen 8‑stelligen Matrikelnummer (MNR) des Studenten (Dateiname „*<Matrikelnummer>*.sav“, z. B. „12345678.sav“).

Der Prüfungsablauf gestaltet sich für die Studierenden wie folgt:

1. Herunterladen Prüfungsdateien (Prüfungsskript und Fragenkatalog)
2. Kompilieren Prüfungsskript
3. Ausführen Prüfungsskript (lädt den Fragenkatalog herein)
4. Starten der Prüfung
5. Beantworten der Prüfungsfragen (erstellt Abgabedateien)
6. Beenden der Prüfung
7. Hochladen Abgabedateien (Antwortdatei und Klartextdatei)

**Figure 1:**
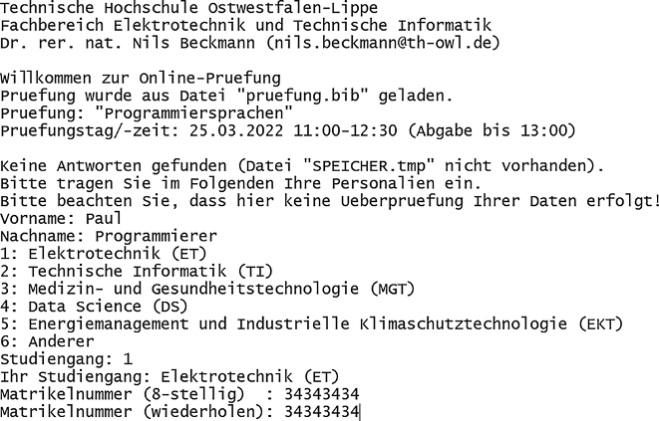

**Figure 2:**
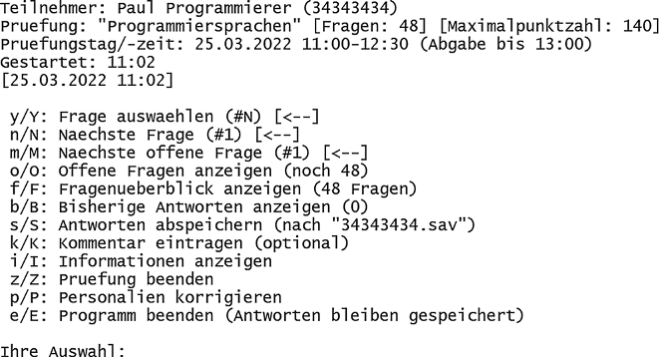

Das Prüfungsskript (Dateiname „pruefung.c“) besteht aus einem Quelltext in der Programmiersprache C, welcher von den Studierenden lokal mit Hilfe weniger Kommandos kompiliert werden muss. Die Wahl der Entwicklungsumgebung ist dabei frei, empfohlen wird die Linux-Emulationsumgebung und Konsolenanwendung Cygwin [16], inklusive des GCC-Compilers [17] mit C11-Standard [11], gegebenenfalls in Verbindung mit NetBeans [18], welches auch in den Praktika Anwendung findet. Das Kompilieren und Ausführen eines C‑Skripts ist somit auch eine notwendige Prüfungsleistung, was in den Praktika vielfach durchgeführt wurde. Die Anzeige des Eingangsbildschirms (Abb. 1) als auch des zentralen Auswahlmenüs (Abb. 2) während der Prüfung sind dargestellt.

**Figure 3:**
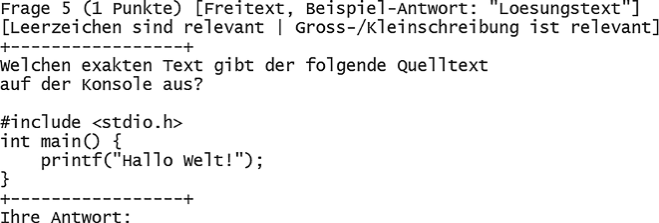

**Figure 4:**
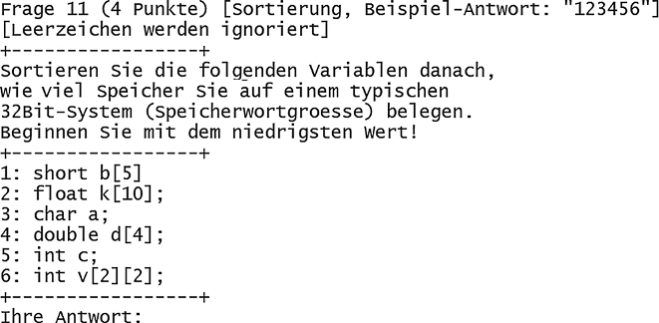

Über den Buchstaben „Y“ kann vom Prüfungsteilnehmer eine Aufgabe zur Bearbeitung ausgewählt werden. 2 Beispielaufgaben sind in den Abb. 3 („Freitext“) und Abb. 4 („Sortierung“) gezeigt. Jede Aufgabe gehört einem bestimmten Aufgabentyp an, der in Tab. 1 dargestellt ist. Alle Aufgabentypen bieten die Möglichkeit, neben automatisierter Vergabe der vollen Punktzahl auch Teilpunkte für teilweise korrekte Antworten zu vergeben.

**Table 1:** 

Aufgabentyp	Punktevergabe
Einzelauswahl („single choice“)	Volle Punktzahl nur bei korrekter Auswahl
Mehrfachauswahl („multiple choice“)	Anteilig Pluspunkte für korrekte Auswahlen und Minuspunkte für inkorrekte Auswahlen
Sortierung	Anteilig Pluspunkte für korrekte Einsortierung und Minuspunkte für inkorrekte Einsortierung
Freitext	Je nach Aufgabe ist die Korrektheit abhängig/unabhängig von Groß‑/Kleinschreibung oder exakter/teilweiser Übereinstimmung
Ganzzahlergebnis	Richtiger Wert gibt volle Punktzahl und suboptimaler Wert kann Teilpunkte geben
Kommazahlergebnis	*(wie zuvor)*
Schätzwert	Punktevergabe je nach Nähe zum korrekten Wert
Rechenergebnis	Punktevergabe innerhalb eines Vertrauensintervalls zum korrekten Wert

Da C eine plattformabhängige Sprache ist, wurden vorab Vorkehrungen getroffen, dass das Prüfungsskript möglichst von allen Prüfungsteilnehmern (mit ihren unterschiedlichen Systemplattformen) eingesetzt werden kann:

- Der C‑Quelltext wurde möglichst plattformunabhängig erstellt und auf verschiedenen Plattformen (= Prozessor + Betriebssystem) vorab umfangreich getestet (Windows 32-Bit und 64-Bit, MacOS, Linux u. a.).
- Das Prüfungsskript wurde bereits lange Zeit vor der Prüfung (Monate vorher) den Studierenden mit einem Fragenkatalog zum selbstständigen Testen bereitgestellt (selbstverständlich enthielt dieser nicht die Prüfungsfragen, sondern Testfragen).
- Ein zusätzliches C‑Skript wurde erstellt (Dateiname „systemtest.c“), welches bei Ausführung diverse prüfungsrelevante Systemtests an der Plattform des Endgeräts des Studierenden durchführte.
- Die letzteren beiden Durchführungen wurden frühzeitig als obligatorisch für die Studierenden bekanntgegeben. Bei möglichen Warn‑/Fehlermeldungen sollten sich die Studierenden rechtzeitig melden, um alternative Lösungen für die technische Durchführung zu finden.
- Zusätzlich wurden diverse Anleitungen zu den Dateien erstellt, darunter enthalten: Hinweisdokument für die Prüfung, Darstellung der Bildschirmanzeigen, Arbeitsanweisung, häufig gestellte Fragen („FAQ“), Informationen zu Prüfung und Systemtest, Erklärvideos.

**Figure 5:**
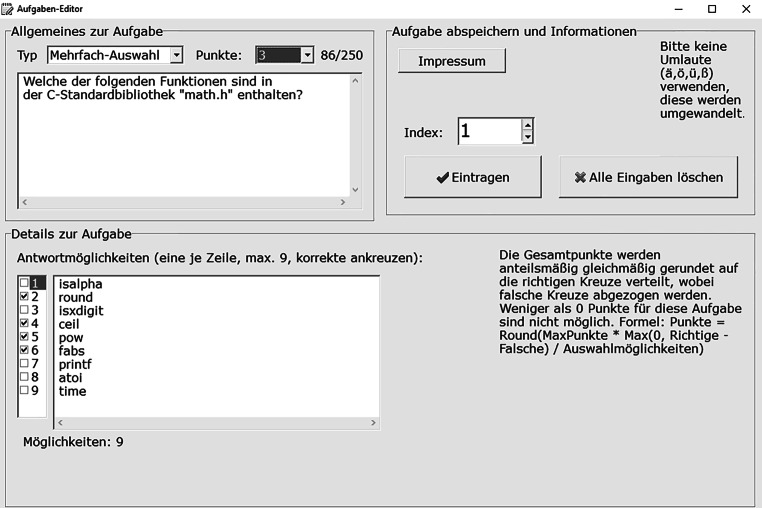

Der Fragenkatalog (Dateiname „pruefung.bib“) enthält Informationen zur Prüfung sowie alle Prüfungsfragen (hier auf maximal 200 Fragen limitiert). Dieser wurde vorab durch ein Verwaltungsskript (Dateiname „verwalter.c“) aus einer Anzahl von Prüfungsaufgaben (je 1 Datei je Aufgabe) zusammengestellt, welche mittels eines in Delphi [19] programmierten Aufgabeneditors mit grafischer Oberfläche erstellt wurden (Abb. 5). Der nur maschinenlesbare Fragenkatalog (exklusive der korrekten Antworten) wird den Prüfungsteilnehmern zu Prüfungsbeginn zur Verfügung gestellt. Über einen Algorithmus wählt das Prüfungsskript automatisiert und randomisiert, abhängig von der Matrikelnummer des Teilnehmers (als „seed“ für den Zufallszahlengenerator), eine Teilmenge der Prüfungsfragen aus und mischt diese durch. Die Anzahl der Fragen kann je Teilnehmer variieren, ist aber durch eine einheitliche, zuvor eingestellte Maximalpunktzahl limitiert. Da Schwierigkeitsgrad und Zeitaufwand für eine Aufgabe durch die dabei erreichbare Punktzahl bemessen sind, erhalten alle Prüfungsteilnehmer durch die Vorgabe einer einheitlichen Maximalpunktzahl Prüfungen mit äquivalentem Anforderungsniveau. Sobald die Prüfung beendet ist (bereits in der Folgeminute), können alle Abgabedateien der Teilnehmer gebündelt aus der ILIAS-Plattform heruntergeladen werden. Das Verwaltungsskript kann diese stapelweise automatisiert einlesen, direkt mittels eines „Vollfragenkatalogs“ korrigieren und einen Notenspiegel der Prüfung erstellen. Der Vollfragenkatalog enthält alle Prüfungsaufgaben (wie der Fragenkatalog), zuzüglich verschiedener korrekter Antwortmöglichkeiten je Aufgabe sowie Punktevergabeschlüssel (auch Teilpunktzahlen sind möglich), und wird vorab ebenso mit Hilfe des Verwaltungsskripts generiert.

Der Prüfungsablauf gestaltet sich für den Prüfer wie folgt:

1. Prüfungsaufgaben erstellen mittels Aufgabeneditor (diese können für Folgeprüfungen wiederverwendet und ausgewählt werden)
2. Prüfungsaufgaben zu Fragenkatalog und Vollfragenkatalog bündeln mittels Verwaltungsskript
3. Prüfungsskript und Fragenkatalog in das ILIAS-Prüfungssystem [9] hochladen
4. Durchführung der Prüfung
5. Abgabedateien (Antwortdatei und Klartextdatei) der Teilnehmer herunterladen
6. Abgabedateien mittels Vollfragenkatalog und Verwaltungsskripts stapelweise korrigieren
7. Manuelle Korrekturen für einzelne Aufgaben vornehmen (insbesondere für Prüfungsaufgaben mit Freitexteingabe, siehe Diskussion)
8. Notenliste mittels Verwaltungsskripts erstellen
9. Prüfungseinsichtsdateien vorbereiten und durchführen

**Figure 6:**
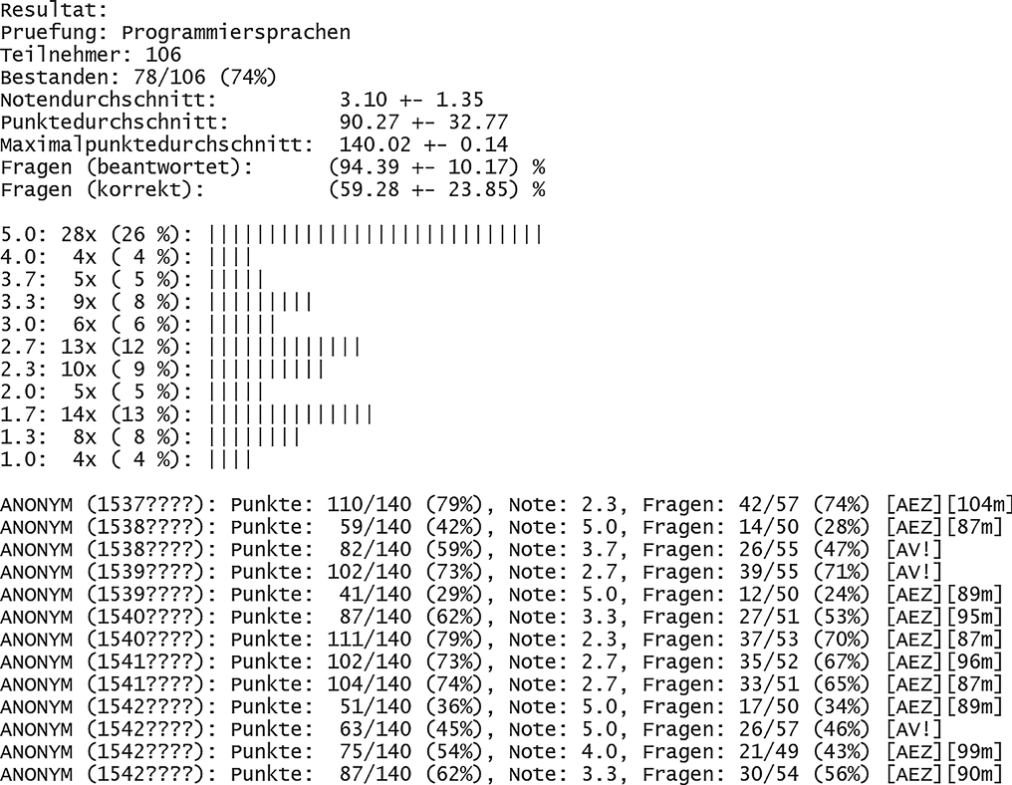

**Figure 7:**
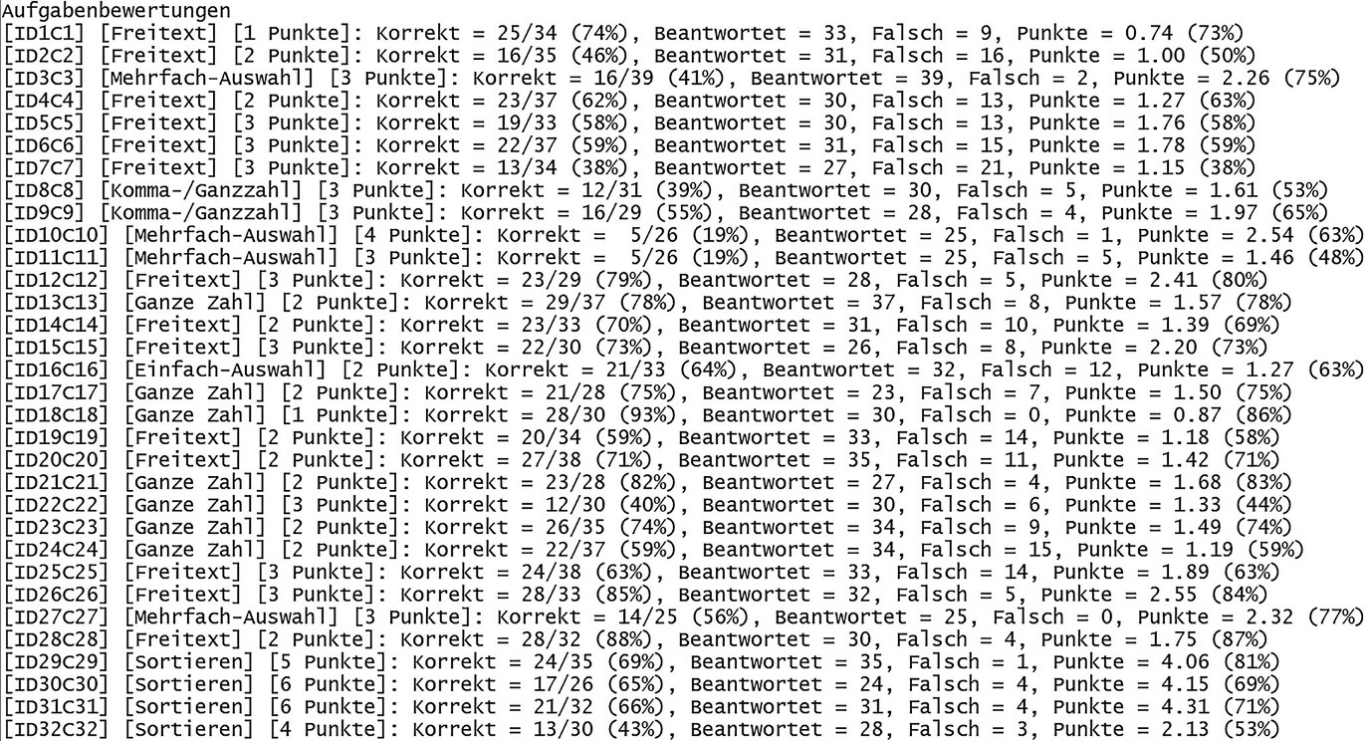

**Figure 8:**
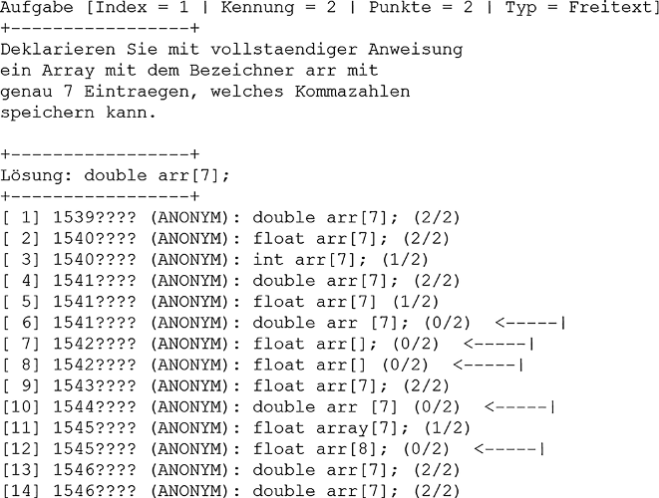

Nachdem alle Abgabedateien nach der Prüfung vom Prüfer heruntergeladen worden sind, können diese stapelweise korrigiert werden, womit alle Punktzahlen sowie Noten der Teilnehmer direkt ablesbar sind (Abb. 6). Auch die Querschnittsergebnisse für alle Aufgaben sind ebenso verfügbar (Abb. 7). Damit ist je Aufgabenthema der jeweilige Stand des Wissenserwerbs bei den Studierenden durch die vorangegangene Vorlesung während des Semesters transparent sichtbar. Typischerweise sind in den vorgefertigten Aufgaben des Vollfragenkatalogs nicht alle möglichen Antworten berücksichtigt, die Punkte erzielen können. Entsprechend ist eine manuelle Nachkorrektur notwendig. Diese wird dadurch vereinfacht, dass die Antworten aller Teilnehmer aufgabenweise gelistet werden können (Abb. 8). Für einzelne Teilnehmer sind die automatisch ermittelten Noten damit noch anzupassen.

Eine zusammenfassende Darstellung der im Vorfeld programmierten Skripte/Softwares (Tab. 2) und notwendigen Dateien (Tab. 3) ist unten gelistet. Die Planung (Spezifikation) und Umsetzung (Implementierung und Qualitätssicherung) sowie Prüfungsdurchführung geschahen durch den Autor.

**Table 2:** 

Skript	Aufgabe	Quelltextzeilen (ca.)	Dateigröße [Kilobyte]	Nutzer
Systemtest („systemtest.c“)	Vorab zur Prüfung der Plattform der Endgeräte der Studierenden	700	22	Student
Prüfungsskript („pruefung.c“)	Zur Durchführung der Prüfung	2700	104	Student
Aufgabeneditor	Zur Erstellung der einzelnen Prüfungsaufgaben	1000	633	Dozent
Verwaltungsskript („verwalter.c“)	Bündelung des Fragenkatalogs sowie Auswertung der Abgabedateien	2700	104	Dozent

**Table 3:** 

Datei	Aufgabe	Dateigröße [Kilobyte]	Ersteller	Nutzer
Fragenkatalog („pruefung.bib“)	Enthält alle Prüfungsaufgaben (hier: maximal 200)	229	Dozent	Student
Vollfragenkatalog („vollpruefung.bib“)	Enthält alle Prüfungsaufgaben inklusive Lösungen (max. 200)	593	Dozent	Dozent
Antwortdatei („*<Matrikelnummer>*.sav“)	Enthält alle Antworten des Prüfungsteilnehmers	29	Student	Dozent
Klartextdatei („*<Matrikelnummer>*.txt“)	Enthält alle Antworten des Prüfungsteilnehmers im Klartext	4	Student	Dozent

### Erfahrungswerte und Diskussion

Studierende sind sich oftmals der Situation bewusst, dass ihr Erfolg bei Absolvierung einer Prüfung von zu Hause aus (in Einzelfällen auch aus dem Ausland) von einer stabilen und schnellen Internetverbindung abhängt, was nicht immer gegeben ist. Entweder sind Fragebögen im Internet mittels eines Webbrowsers durchzuführen oder zu Prüfungsende sind abfotografierte oder gescannte Schriftstücke mit erheblicher Datengröße (2-stellige Megabyte) hochzuladen, was bei hohen Teilnehmerzahlen bei Prüfungen zu serverseitigen Verzögerungen führt. Die Eintragung oder Abgabe von Antworten kann dadurch verzögert oder sogar verhindert werden. Diese Hürden werden durch die hier vorgestellte Prüfungsform (weitestgehend) entfernt, da lediglich zu Beginn und am Ende der Prüfung kleine Datenmengen (insgesamt weniger als ein halbes Megabyte, Tab. 3) zu übertragen sind. Dadurch wird auch Studierenden mit qualitativ schlechterer Internetverbindung eine Prüfungsteilnahme ermöglicht.

Die vorgestellte Prüfungsform wurde bereits für mehrere Prüfungen angewandt. Typischerweise bekamen die Prüfungsteilnehmer automatisiert um die 30–50 Aufgaben je Prüfung zugewiesen, was bei 90 min Prüfungszeit folglich rund 2–3 min Bearbeitungszeit je Aufgabe ermöglicht. Die Fragenkataloge bestanden aus 70 bis zu 180 Aufgaben, aus denen der Algorithmus ausgewählt hat. Je Aufgabe konnten zwischen 1 und 8 Punkte erreicht werden. Die Maximalpunktzahl betrug üblicherweise 120 oder 140 Punkte. Diese Form der Prüfung wurde nicht nur im Modul „Programmiersprachen 1“ (C-Programmierung) durchgeführt, sondern auch in höheren Semestern und weiteren Modulen („Programmiersprachen 2“ für Java-Programmierung, „Software Lifecycle Management“ sowie „Alltagsphysik“ mit Rechenaufgaben). Das selbstständige Kompilieren und Ausführen des Prüfungsskriptes ist jedes Mal integraler Bestandteil der Prüfung und auch Prüfungsleistung, was für die C‑Programmierung selbstverständlich ist – in anderen Modulen jedoch nicht – und daher frühzeitig bekanntgegeben und durch schriftliche Dokumentationen (siehe oben) unterstützt werden muss. Bei Studierenden höherer Fachsemester ist der Prüfungsdurchlauf in der Regel problemlos, wohingegen Erstsemester oder fachlich schwächere oder unvorbereitete Studierende in Einzelfällen (weniger als 5 %) selbstverschuldete Probleme mit dem Prüfungsskript haben: Entweder haben diese nicht Systemtest oder Testskript laufen lassen oder sind während der Prüfung nervös und scheitern dadurch bereits am Kompilierungsvorgang (Navigieren in den Ordner mittels Konsole schlägt fehl, Kompilierungsbefehl wird im falschen Ordner ausgeführt, Compiler und/oder Cygwin ist nicht installiert, verspätete Abgabe wegen Nutzung der Pufferzeit für das Beantworten von Aufgaben und so weiter). Hierzu wurde während der Prüfung in einem Online-Raum mittels der Software „Adobe Connect“ [20] und Bildschirmfreigabe für diesen ersten Schritt von Aufsichtspersonen rudimentäre Unterstützung gegeben. Da diese Studierenden aber ohnehin in der Regel wenig Lernvorbereitung hatten, erreichten sie die erforderliche Punktzahl zum Bestehen üblicherweise nicht.

Die Verhinderung von Täuschungsversuchen und Betrug bei Online-Prüfungen ist ein Thema für sich [21, 22] und betrifft auch die hier vorgestellte Prüfungsform. Teilnehmer können unerlaubte Hilfsmittel benutzen, wie beispielsweise das Internet, sich von anderen Menschen im selben Raum (zu Hause) beraten lassen oder sogar die gesamte Prüfung von Kontakten irgendwo auf dem Planeten absolvieren lassen. Entsprechende Forschungsaktivitäten versuchen [8, 23], die Möglichkeiten dafür einzudämmen, beispielsweise mittels Kameraüberwachung oder anderer Formen der digitalen Protokollierung. Auch Studierenden sind die Möglichkeiten zur Täuschung bewusst und diese wünschen sich gerechte Prüfungsformate [7]. Für die vorgestellte Methode bewirken die erwähnte Randomisierung sowie Mischung der Prüfungsaufgaben eine Reduktion der Täuschungsmöglichkeiten, da Antworten nicht oder nur nach vorheriger zeitaufwändiger Suche und Prüfung der korrekten Aufgabe von einem Teilnehmer zum anderen übertragen werden können. Zudem wurde den Teilnehmern eine Vielzahl an Hilfsmitteln gestattet (Fachbücher, Vorlesungsunterlagen, Quelltexte, Handnotizen, Übungszettel usw.), ähnlich einer „Open-Book“-Klausur, so dass der Vorteil durch eine unzulässige Erweiterung der Hilfsmittel (z. B. Nutzung des Internets, Kommilitonen fragen) geringer ausfällt. Weiterhin ist die Zeit pro Frage (im Vergleich zu Papierklausuren) durch Erhöhung der Fragenanzahl (jedoch mit kürzeren Antworten je Frage) etwas reduziert worden, damit weniger Zeit für mögliche betrügerische Aktivitäten bleibt. Die Fragenanzahl und damit der Schwierigkeitsgrad der Prüfung ist durch Variation der Maximalpunktzahl auf einfache Weise stufenlos einstellbar.

Der zeitliche Aufwand zur Korrektur der Prüfungen teilt sich auf in die automatisierte und manuelle Korrektur. Erstere ist hier in wenigen Minuten für beliebige Teilnehmerzahlen möglich. Letztere benötigt mehr Zeit und betrifft insbesondere Aufgaben vom Typ „Freitext“ sowie „Rechenergebnis“ (Tab. 1). Bei freier Texteingabe können beispielsweise Rechtschreibfehler auftreten, Eingabe von Sonderzeichen oder überflüssige/fehlende Leerzeichen oder die Eintragung anderer sinngemäß (teilweise) korrekter Antworten. Bei Rechenergebnissen können beispielsweise Vorfaktoren vergessen worden sein, das Ergebnis wurde in einer falschen physikalischen Einheit angegeben oder es wurde ein Ergebnis berechnet, welches die Vergabe von Teilpunkten rechtfertigt. Gemäß Erfahrungswerten dauert die Korrektur einer gesamten digitalen Prüfung für eine Person grob 2 h bei Teilnehmerzahlen um die 100 (Abb. 6). Je Teilnehmer kann man (zusätzlich basierend auf weiteren Prüfungserfahrungen) folglich etwa 1 min Zeitaufwand einrechnen, inklusive einer Sockelzeit um die 20 min. Bei klassischen Präsenzklausuren beträgt der Korrekturaufwand (bei stringent strukturierter Korrekturweise, z. B. aufgabenweise) typischerweise um die 10 min je Teilnehmer, womit dieser durch das hier vorgestellte Format um bis zu 90 % reduziert werden kann. Eine automatisierte Punktevergabe funktioniert i. d. R. nur, sofern Antwortmöglichkeiten, welche Punktevergabe erfordern, vorhersehbar sind. Dies betrifft selbstverständlich korrekte Antworten und darüber hinaus auch die verschiedenen suboptimalen Lösungsmöglichkeiten (Abb. 8). Wie angesprochen ist eine manuelle Nachkorrektur insbesondere für Aufgaben vom Typ „Freitext“ notwendig, weil die automatisierte Korrektur mit der Länge und Komplexität von optimalen Lösungen schwieriger wird. Entsprechend wurden, im Rahmen der hier vorgestellten Prüfungen, Lösungsantworten kurzgehalten sowie die Eingabe von Teilnehmerantworten auf eine Zeile limitiert. Dadurch entsteht der Nachteil, dass keine längeren Texteingaben als Antworten möglich sind, wodurch beispielsweise das Programmieren von (längeren) Quelltexten oder Quelltextbausteinen ausgeschlossen ist (Abb. 3 und Abb. 8). Aufgaben mit Bezug zu mehrzeiligem Quelltext, was in Papierklausuren üblich ist, müssen diesen entsprechend in die Aufgabenstellung mit integriert haben (Abb. 3 und Abb. 4). Trotz dieser Einschränkung sind die Notenspiegel von Papierklausuren sowie dem hier vorgestellten Format, gemäß den gemachten Erfahrungen (siehe oben), durchaus vergleichbar [8]. Für Klausureinsichten wird mittels des Verwaltungsskriptes eine Ausgabedatei je Teilnehmer erzeugt, welche dessen gegebene Antworten sowie die vergebene Punktzahl enthält (wahlweise kann auch eine korrekte Lösung mit integriert werden). Diese können den Teilnehmern entweder gedruckt, am Beamer oder durch digitale Bildschirmfreigabe präsentiert werden.

Zusammengefasst ist die hier vorgestellte digitale fragebogenbasierte Prüfungsform über ein Programmierskript gut durchführbar, wurde in der Praxis demonstriert und ermöglicht Prüfungsdurchführungen auch für Teilnehmer mit langsamen oder instabilen Internetverbindungen. Der Korrekturaufwand kann gegenüber papierbasierten Klausuren erheblich reduziert werden und die Notenspiegel fallen ähnlich zu denen bisheriger papierbasierter Klausuren aus.
